# Psychiatric Sequelae of Dengue: A Review of the Interface

**DOI:** 10.1155/jotm/7136558

**Published:** 2025-05-25

**Authors:** Priyanka Renita D'Souza, Debora Sona D'Silva

**Affiliations:** ^1^Department of Psychiatry, Kasturba Medical College Mangalore, Manipal Academy of Higher Education, Manipal, India; ^2^Department of Medicine, Father Muller Medical College, Mangalore, Karnataka, India

## Abstract

Dengue is one of the major public health concerns in tropical and subtropical countries. In addition to neurological sequelae which are well documented, emerging evidence suggests that dengue may also lead to psychiatric sequelae including mood disorders, psychosis, anxiety, and body dysmorphic disorder. This narrative review aims to synthesize the existing literature to explore the psychiatric manifestations and postulated pathophysiological mechanisms and identify predictors and treatment of psychiatric sequelae in dengue. This review identified 30 studies including observational studies, case reports, and case series. The immune-inflammatory responses due to cytokine dysregulation, blood–brain barrier disruption, direct viral effects, and epigenetic mechanisms with histone deacetylase activation are possible contributors to psychiatric sequelae in dengue. The main predictors include severity of dengue, thrombocytopenia, central nervous system involvement, febrile and critical phase of illness, specific dengue virus serotypes (DENV-2 and DENV-3), and stress due to hospitalization. Psychiatric symptoms often persist beyond the acute phase, highlighting the importance of long-term follow-up to evaluate the impact of dengue on mental health. Additionally, comparisons with other Flaviviridae viruses, such as Zika, West Nile, and Japanese encephalitis viruses, reveal both shared and distinct psychiatric implications, suggesting potential virus-specific mechanisms. The current treatment approaches are largely extrapolated from general psychiatric practice, with limited research on targeted interventions. Future research should focus on standardized diagnostic assessment, longitudinal follow-up, diagnostic biomarkers, and developing targeted treatment strategies to improve clinical outcomes. With rising cases of dengue, integrating psychiatric screening into routine dengue management may enhance early recognition and intervention. Hence, a multidisciplinary research approach involving psychiatrists, neurologists, infectious disease specialists, immunologists, and policymakers is crucial for addressing psychiatric sequelae in dengue fever and mitigating the detrimental implications on public health.

## 1. Introduction

Dengue also called 7-day fever or breakbone fever, caused by dengue virus (DENV), an arthropod-borne virus, transmitted by *Aedes aegypti* is one of the most important public health concerns [1]. Dengue fever may have a wide spectrum of symptoms, ranging from mild flu-like symptoms to dengue hemorrhagic fever (DHF) and dengue shock syndrome (DSS) which has a high morbidity and mortality rate [2]. Five DENV serotypes (DENV-1 to DEN-5) were identified based on antigenic differences with serotypes DENV-2 and DENV-3 predominantly associated with neurological manifestations [3, 4]. Neurological presentation associated with dengue encompassing encephalitis, myelitis, meningitis, Guillain–Barré syndrome, and stroke are increasingly reported, but the psychiatric sequelae are poorly understood [5]. Dengue fever may contribute to a range of psychiatric manifestations including mood disorders, psychosis, and anxiety symptoms [6–35].

The course of dengue fever has three phases characterized by febrile, critical, and recovery phases. The critical period is an important phase in terms of the severity of the illness and may cause neurological, hepatic, and cardiac dysfunction [36], but there has been limited research on psychiatric complications. Despite the case reports and observational studies suggesting a link between dengue and psychiatric manifestations, the exact pathophysiological mechanisms, predictors, and long-term outcomes remain unclear [6–35]. The heterogeneity in study designs and outcome measures highlighted the need for a narrative review. Hence, the primary aim of this narrative review is to present an integrated overview of the existing literature on psychiatric manifestations associated with dengue fever and thus identify the knowledge gap with the objective to the following:

• Assess the association between dengue and psychiatric manifestations
• Explore the pathogenesis underlying the association
• Identify the predictors of psychiatric sequelae in dengue
• Map the current literature on the management of psychiatric sequelae in dengue

## 2. Methods

The methodological approach for this review does not strictly adhere to the classical systematic review framework, but we have followed the Preferred Reporting Items for Systematic Reviews and Meta-Analyses (PRISMA) guidelines [37] wherever applicable to ensure methodological transparency and rigor.

### 2.1. Search Strategy

A thorough literature search was conducted in August 2024 using the Embase, PubMed, and Scopus databases. The search strategy included the following key terms: “Dengue” AND (“Depression” OR “Anxiety” OR “Mania” OR “Bipolar disorder” OR “Psychosis” OR “Schizophrenia” OR “Catatonia” OR “Obsessive-Compulsive Disorder” OR “Suicide” OR “Psychiatry” OR “Mental health”).

The selection of studies was guided by the Population, Exposure, Outcome (PEO) framework, ensuring a structured and systematic approach (Table 1).

### 2.2. Eligibility Criteria

The inclusion and exclusion criteria for study selection were established as mentioned in Table 2.

Two independent reviewers conducted a title and abstract screening, followed by a full-text review of eligible studies. The selection process is mapped in the Supporting File.

A total of 30 studies were included in this review [6–35], comprising the following:

• 14 observational studies,
• 14 case reports, and
• Two case series.

A review was conducted, and the results were categorized into relevant domains and summarized in Tables 3, 4, 5, and 6.

## 3. Results and Discussion

### 3.1. Psychiatric Manifestations in Dengue

An increasing number of studies suggest a link between dengue and psychiatric disorders; however, exact prevalence estimates cannot be determined due to the limited number of observational studies and the predominance of isolated case reports or series, which lack generalizability. The detailed review of the existing research showed a broad range of psychiatric sequelae following dengue fever, which were grouped into the following categories: depression and anxiety, mania, psychosis, and body dysmorphic disorder.

#### 3.1.1. Depression and Anxiety

Depressive and anxiety symptoms frequently co-occurred in the studies examining psychiatric sequelae of dengue. A higher prevalence of depressive symptoms was associated with dengue, particularly during the acute phase of illness. Cross-sectional and prospective studies [6–8, 10] found that approximately 60%–81% of individuals experienced depressive symptoms during the acute phase. Although symptoms were often transient, follow-up assessments [6, 15, 16] observed residual depressive symptoms in a few of them even up to 3 months postinfection. Postdengue depressive symptoms were also reported in 15%–34% of patients [11, 12], persisting up to 24 months. Larger retrospective cohort studies [17, 19] observed elevated risk for mood disorders following dengue; however, methodological limitations, particularly regarding clinical characteristics, severity of dengue, and symptom onset raise concerns about potential confounding factors, including pre-existing psychiatric conditions.

Anxiety symptoms were frequently observed during the acute phase of dengue, with the prevalence ranging from 60% to 80% [6–8]. Thanatophobia and insect phobia were also noted, especially in the early course of dengue fever [6, 9]. Anxiety symptoms typically subsided within weeks, but in certain cohorts [14, 16], symptoms persisted into recovery. Retrospective studies [12, 17, 18] confirmed an elevated risk of anxiety disorders in the postdengue phase, although the study by Shih et al. [19] found no similar association.  Table 3 gives an elaborate description of the studies with depression and anxiety in the acute phase as well as later stages of dengue.

#### 3.1.2. Mania

All the studies included in this review that reported features of mania after dengue fever were case reports [20–23, 25–27], except for one case series [24]. Mania after dengue infection was typically observed during the acute or febrile phase and shortly after recovery. Onset ranged from the third day of illness to within 2 weeks postrecovery, with some cases occurring after the resolution of fever.  Table 4 elaborates on the studies with mania following dengue fever.

#### 3.1.3. Psychosis

All the studies reporting psychotic features after dengue infection were case reports [28–34], except one large retrospective cohort study by Lin et al. [17], as described in Table 5. Psychotic symptoms, including agitation, delusions, and auditory and visual hallucinations were observed during both acute and recovery phases. Catatonia was also noted in one of the case reports [29]. In a few cases, psychotic features were associated with dengue encephalitis or DSS, which complicated the diagnostic picture [33, 34]. The retrospective cohort study found an increased risk of psychotic, mood, and anxiety disorders after dengue, although the prevalence of individual psychiatric disorders and clinical data were not provided, making it challenging to ascertain the exact prevalence of postdengue psychosis [17].

#### 3.1.4. Body Dysmorphic Disorder

There was only one study that reported an association between dengue and body dysmorphic disorder [35]. Although it may seem rare, awareness among clinicians is necessary. Significant hair loss occurring approximately after 1 month of dengue was an important factor in the progression of body dysmorphic disorder.

Interestingly, a Brazilian study described the inverse relationship between dengue and psychiatry. The study found that compulsive hoarding, particularly the accumulation of trash and waste, may pose a public health risk by creating an environment conducive to mosquito breeding and the spread of dengue [38].

### 3.2. Pathogenesis

Despite its growing significance, etiological mechanisms underlying psychiatric manifestations in dengue are not extensively studied. However, recent evidence suggests psychiatric sequelae in dengue could be due to the dynamic interaction of inflammatory, immune-mediated mechanisms, and epigenetics.

Viral illness such as dengue triggers an inflammatory response with an increase in proinflammatory cytokines such as interleukin-1β (IL-1β), interleukin-2 (IL-2), interleukin-6 (IL-6), and TNF-alpha. These cytokines cause the disruption of the blood–brain barrier promoting neuroinflammation and neuropsychiatric manifestations. Observational studies reported a higher prevalence of depressive and anxiety symptoms during the febrile phase of dengue fever, suggesting a cytokine-mediated link for psychiatric sequelae [8, 13, 14, 16, 19].

**Figure 1:**
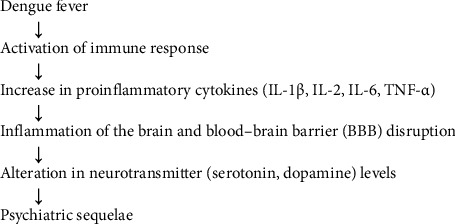
Inflammatory pathway linking dengue infection to psychiatric sequelae.

Neurotransmitter dysregulation, particularly altered serotonin levels, has also been proposed as an etiological factor. Serotonin, an important neurotransmitter involved in mood regulation, is known to be influenced by the neurotoxic inflammation in dengue fever. This inflammation due to dengue infection may alter serotonin metabolism, potentially leading to mood disorders such as depression and anxiety, as described in Figure 1. Inflammatory cytokines released during dengue fever may decrease tryptophan levels and affect serotonin synthesis, contributing to depressive symptoms [19].

**Figure 2:**
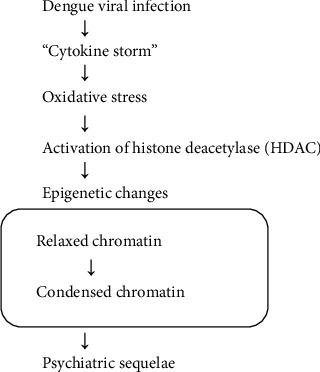
A proposed epigenetic mechanism linking dengue infection to psychiatric sequelae.

Epigenetic mechanisms, specifically histone deacetylase (HDAC) activation, have been implicated in the pathogenesis of psychiatric disorders related to dengue. DENV triggers a “cytokine storm,” leading to oxidative stress and the subsequent activation of HDAC. HDAC activation induces epigenetic changes, which may alter gene expression related to mood regulation and neuronal function, as described in Figure 2. The case report by Dinakaran et al. discusses postdengue mania highlighting the etiological mechanism of HDAC and suggesting a potential therapeutic role for HDAC inhibitors [25].

DENV modifies host metabolism to support its replication, interacting with cellular genes and long noncoding RNAs (lncRNAs) to induce m6A RNA methylation, which enhances viral propagation. This mechanism is shared across the Flaviviridae family, including West Nile, yellow fever, and Zika viruses, as they also exhibit m6A methylation, though each flavivirus has a unique methylation pattern [39].

Psychiatric manifestations are not exclusive to dengue but are also observed in other arboviral infections, highlighting shared pathophysiological mechanisms. Zika virus, due to its neurotropism, has been associated with bipolar disorder, schizophrenia, autism, and attention deficit hyperactivity disorder [40]. Chikungunya virus is associated with postviral depression, anxiety, and fatigue, likely due to CNS inflammation and cytokine overproduction [41]. Similarly, West Nile virus, through cytokine dysregulation and blood–brain barrier disruption, has been implicated in depression, chronic fatigue, and psychosis [42, 43]. These findings suggest that neuroinflammation, cytokine dysregulation, and direct viral invasion contribute to the psychiatric effects of arboviral infections.

### 3.3. Predictors of Psychiatric Sequelae of Dengue

A. The clinical parameters include the following:
  - High-grade fever, intense headache, and severe myalgia have a positive correlation with depressive and anxiety symptoms [8, 12, 14, 16] and were also predominantly noted with manic and psychotic symptoms.
  - A possible hypothesis is that headache and pain contribute to elevated inflammatory cytokine levels [8, 13, 14, 16, 19].
B. The phase of illness includes the following:
  - Psychiatric symptoms are reported across all clinical phases of dengue but are more prevalent in the febrile and critical phases of dengue.
  - Symptoms may emerge due to cytokine surges and systemic inflammation in the febrile phase and vascular permeability and immune dysregulation in the critical phase [8, 13, 14, 16, 19].
C. The severity of dengue is discussed as follows:
  - Less consistent association between psychiatric sequelae and DHF or DSS.
  - However, mania has been linked in the context of DHF in a case report [26], and Mushtaq et al. observed elevated depression, anxiety, and stress scores in patients with DHF and DSS [10]. A case-control study in a pediatric population found a higher prevalence of psychiatric symptoms (76.4%) in hospitalized children with DHF [14]. DSS was also reported in a few cases with subsequent manic or psychotic symptoms [10, 26, 33].
  - These associations are possibly due to cerebral hypoperfusion and edema, direct tissue lesion, and metabolic disturbances.
D. Thrombocytopenia includes the following:
  - Another prominent finding was the strong association between thrombocytopenia and psychiatric manifestations in the observational studies [8, 12, 20, 21].
  - Potential mechanisms include immune response activation, hypoxia-related neuronal stress, or psychological reaction to the severity of dengue [8].
E. The central nervous system (CNS) involvement includes the following:
  - The presence of encephalitis, encephalopathy, altered sensorium, or seizures suggestive of CNS involvement increases the risk of psychiatric sequelae [9, 20, 21, 26, 28, 34].
  - Case reports have documented CNS involvement in patients with secondary mania and psychosis associated with dengue fever, further substantiating its potential role in psychiatric manifestations [9, 20, 21, 26, 28, 34].
  - The underlying pathogenesis of encephalitis and encephalopathy may result from direct viral invasion of the brain, cerebral edema, and vascular hypoperfusion [44].
F. DENV serotype includes the following:
  - DENV-2 and DENV-3 serotypes are responsible for neurological complications due to neurovirulence. Only one study in the review identified the serotype and found that DENV-3 was linked with neuropsychiatric manifestations [18].
  - Animal models suggest DENV-3 infection causes anxiety-like behavior, neuronal apoptosis, and hippocampal inflammation [45].
G. Psychological distress from illness includes the following:
  - Shih et al. attribute depressive and anxiety symptoms to acute stress reactions triggered by dengue-associated symptoms and hospitalization, particularly in severe cases [19].
  - The development of body dysmorphic disorder was linked to dengue-associated hair loss, often a result of telogen effluvium, suggesting that postdengue physical changes may contribute to psychological distress [35].
  - Unanticipated disruptions in life and insomnia can exacerbate mental health disorders [19].

However, the exact nature of the mechanisms influencing psychiatric manifestations is yet to be elucidated.

In addition, retrospective studies explored the long-term psychiatric outcomes of dengue fever, highlighting persistent psychiatric manifestations beyond the acute phase of illness. These studies identified prolonged and delayed symptoms such as depression, stress, anxiety, and sleep disorders, suggesting that dengue may have enduring effects on mental health [12, 17–19]. The findings indicate that long-term psychiatric complications following dengue may arise from factors independent of the immediate stress of hospitalization or physical illness.

### 3.4. Management of Psychiatric Sequelae of Dengue

There are no approved antiviral drugs to treat dengue fever, and the primary management is predominantly symptomatic. Corticosteroids are considered in DSS and are also sometimes preferred by clinicians to prevent disease progression to severe form [46]. Notably, corticosteroids are associated with myriads of psychiatric disturbances including mania, depression, and psychosis [47]. However, the primary medical management of dengue fever with specific pharmacological interventions has not been well explored in the literature to establish a clear temporal relationship with psychiatric sequelae. Distinguishing whether psychiatric sequelae arise from dengue itself or as a consequence of corticosteroid use poses a significant challenge. The overlap in psychiatric manifestations, such as mood disturbances, psychosis, and anxiety symptoms, complicates the identification of the primary etiological factor. Furthermore, the variability in individual susceptibility to corticosteroid-induced psychiatric effects adds another layer of complexity. The absence of systematic studies examining the temporal onset of psychiatric symptoms to corticosteroid administration further hinders clarity, emphasizing the need for well-structured research in this domain.

The management of depressive and anxiety symptoms in dengue remains underreported as the observational studies in the review did not include pharmacological or psychological interventions [8, 12, 14, 17–19]. However, Gill et al. [6] noted that while symptoms were largely transient, short-term anxiolytics were used in a few cases, and Uvais et al. [13] reported a small subset receiving psychotropics based on their discharge summaries.

Secondary mania due to dengue is effectively managed with mood stabilizers such as valproate, carbamazepine, and antipsychotics such as haloperidol, risperidone, olanzapine, and quetiapine [13, 20–22, 24–28, 30–33]. HDAC inhibitors such as valproate and quetiapine, commonly used in the treatment of bipolar affective disorder, have shown therapeutic benefits in managing dengue-associated mania, reinforcing the etiological role of HDAC-mediated processes [25]. Interestingly, the role of alpha 2 adrenergic receptor agonists is explored as an adjunct treatment in secondary mania associated with dengue encephalopathy. Centrally acting alpha 2 adrenergic receptor agonists such as clonidine, an antihypertensive, and tizanidine, a spasmolytic, were used in the treatment of mania and with better tolerability in patients vulnerable to extrapyramidal symptoms [26]. Psychotic symptoms secondary to dengue are managed with antipsychotics [28, 30–34], whereas catatonia associated with dengue fever has shown improvement with lorazepam [29]. Additionally, body dysmorphic disorder, a rare psychiatric sequelae to dengue-associated telogen effluvium has a favorable response to sertraline and clonazepam [35].

### 3.5. Challenges and Future Directions of Research

Several limitations exist in the current evidence of the literature on psychiatric sequelae of dengue. Although few studies utilized standardized psychiatric assessment tools, their application in the context of dengue remains limited [8, 12, 14, 15, 22]. These tools are primarily designed for general psychiatric screening and may not adequately screen for dengue-specific psychiatric sequelae. The heterogeneity in methodology ranging from structured clinical interviews to self-report scales highlights the need for standardized, longitudinal studies. Furthermore, the lack of consistent diagnostic criteria across the studies makes it challenging to establish the prevalence and severity of psychiatric manifestations associated with dengue.

The potential confounding factors such as family history of psychiatric illness, personality traits, and substance use were not accounted for in any of the observational studies, which may influence the reported findings. However, Gill et al. [6] excluded individuals with a prior history of psychiatric illness, while Uvais and Moideen [13] reported comorbid psychiatric conditions, including depressive disorder and autism spectrum disorder. Physical comorbidities were noted in a few of the observational studies [12, 17], but their role in psychiatric outcomes remains unclear. Most of the studies on psychiatric manifestations of dengue were case reports, and hence, the inferences derived may seem anecdotal.

The lack of large-scale, prospective studies suggests that psychiatric sequelae of dengue may be underreported, partly due to a lack of awareness among clinicians. Furthermore, long-term psychiatric outcomes in dengue remain poorly studied, with only a few observational studies [12, 17–19] addressing this aspect. Clinical trials should assess whether early psychiatric interventions including psychotropics and psychotherapy can improve long-term outcomes. Another notable knowledge gap is the absence of studies examining a potential association between suicide and dengue infection. One of the observational studies found an association between substance use and dengue infection, but the influence of preexisting psychiatric disorders or psychosocial stressors on this relationship was not explored [17].

Neuroimaging findings were unremarkable in most of the cases [9, 21, 22, 27, 29–34], and cerebrospinal fluid (CSF) analysis in cases with dengue encephalitis was normal [9, 34]. However rigorous research utilizing advanced neuroimaging techniques such as functional MRI (fMRI), diffusion tensor imaging (DTI), and positron emission tomography (PET) scans could elucidate neuroinflammatory and structural changes associated with psychiatric manifestations in dengue.

In addition, the role of molecular and genetic factors in secondary psychiatric manifestations remains largely unexplored. Further studies are needed to evaluate the proposed pathophysiological mechanisms, including HDAC activation in the psychiatric sequelae of dengue. Epigenetic studies and genetic susceptibility markers may provide an understanding of the long-term psychiatric outcome of dengue. Furthermore, biomarker-based research should focus on diagnostic markers of inflammatory diseases such as CSF CXCL-10, which has proven high specificity and sensitivity for neuroinvasive DENV infection [48]. Identifying similar biomarkers for psychiatric sequelae could help in early diagnosis and targeted interventions.

To bridge this gap, future research should focus on large-scale, longitudinal studies, standardized psychiatric assessments, and integration of biomarkers with advanced neuroimaging to determine potential therapeutic targets and thus improve clinical outcomes.

## 4. Conclusion

Despite the substantial impact on mental health and overall quality of life, psychiatric manifestations of dengue have garnered little attention. Hence, a multidisciplinary research approach involving psychiatrists, neurologists, infectious disease specialists, and immunologists is crucial for addressing psychiatric sequelae of dengue fever. If untreated, they can lead to increased mortality and morbidity, healthcare utilization, and economic burden. Hence, it is imperative to educate both clinicians and patients about psychiatric complications of dengue. Screening for psychiatric disorders in dengue infection and incorporating standardized tools for assessment in treatment protocols of dengue may help in comprehensive patient care.

## Figures and Tables

**Table 1:** PEO framework.

Population (P)	Individuals diagnosed with dengue
Exposure (E)	Dengue fever
Outcome (O)	Psychiatric manifestations, including but not limited to depression, anxiety, mania, bipolar disorder, psychosis, schizophrenia, catatonia, obsessive-compulsive disorder, and suicidal behavior

**Table 2:** Eligibility criteria.

Inclusion criteria	Exclusion criteria
- Studies examining the association of dengue and psychiatric manifestations	- Review articles
- Original research articles (observational studies, case reports, case series)	- Commentaries/opinions
- Studies published from inception to 2024	- Conference proceedings
- Studies published in peer-reviewed journals	- Animal studies
- English language	

**Table 3:** Depression and anxiety secondary to dengue infection—key findings include a high prevalence of depressive and anxiety symptoms in the acute phase of dengue.

Reference	Type of study	Psychiatric manifestation in dengue	Onset of psychiatric symptoms	Remarks
Gill et al. [6]	Prospective study (*n* = 119)	- Acute phase: Anxiety (∼80%) and depressive symptoms (60.5%), thanatophobia (∼90%) - Within 1 week: Anxiety (31.93%) and depressive symptoms (18.48%), thanatophobia (50%) - After 6 weeks: Anxiety (1.68%), thanatophobia (5.8%) - After 3 months: Mild to moderate depressive episode (5%), anxiety, and thanatophobia resolved		Psychiatric assessment conducted by the psychiatrist using ICD-10 diagnostic criteria Insect phobia was noted after the recovery (56.30%) Symptoms were mostly transient; few required short-term anxiolytics No cases of psychosis or mania Emphasis on psychological support during early phases of illness Limitation: Short follow-up duration
Khan et al. [7]	Cross-sectional (*n* = 97)	Depression: 81.5% Anxiety symptoms: 66%	Acute phase	Limitation: Hospital anxiety and depression Scale (HADS) used; possible bias due to self-reporting
Hashmi et al. [8]	Cross-sectional (*n* = 531)	Depressive symptoms: 62.2% Anxiety symptoms: 60%	Febrile—critical phase of dengue	Predictors of depression and anxiety: Fever, headache, retroorbital pain, myalgia (*p* < 0.01 for all), thrombocytopenia (*p* < 0.01 for depression and *p* < 0.03 for anxiety) Limitations: - The sample size was limited to in-patients - Nonspecific rating scale hospital anxiety and depression scale (HADS) were utilized
Mamdouh et al. [9]	Case series	Anxiety, thanatophobia	Acute phase	Both cases had features of dengue meningitis. Neuroimaging and cerebrospinal fluid analysis were normal except for dengue IgM positive in both cases and pleocytosis in Case 1 Associated features: Headache, retroorbital pain, arrhythmia, thrombocytopenia
Mushtaq et al. [10]	Cross-sectional (*n* = 200)	High scores of depression, anxiety, and stress scores with DHF and DSS were observed, negatively affecting self-efficacy	Acute phase	Thirty-one percent had dengue hemorrhagic fever and 12% dengue shock syndrome Limitation: DASS was administered, but specific parameter scores were not mentioned
Ali et al. [11]	Cross-sectional (*n* = 50)	Depression (34%)	Postdengue	Diagnosis as per DSM IV criteria Limitation: The exact duration of “post-dengue” was not well defined in terms of time
Gunathilaka et al. [12]	Case-control (*n* = 53 in each group)	Higher depressive, anxiety, and stress scores. Depressive disorder: 15.1%	6–24 months postdengue	Predictors of depression, stress, anxiety: Thrombocytopenia (*p* value is 0.038 and 0.010, respectively, with CESD 20 and DASS 21 depressive scores) Structured clinical interview by psychiatrists in addition to standard scales such as CESD 20, and DASS 21 Limitation: Small sample size
Uvais et al. [13]	Retrospective (*n* = 245)	Adjustment disorder (14.30%)	Acute phase	Discharge summary, psychiatric consultation, and psychotropic prescription were reviewed Comorbid depressive disorder (7.10%) and autism spectrum disorder (7.10%) was noted. About 5.71% of patients received psychotropics (quetiapine, clonazepam, sertraline, amitriptyline), but only four patients had a detailed psychiatric evaluation
Herbuela et al. [14]	Case-control (cases = 225, controls = 260)	Depressive symptoms: 13.3% Anxiety symptoms: 34.2%	Acute and recovery phases of dengue	Study sample: Pediatric in-patients Majority were in the acute phase (79.6%), and 76.4% had dengue hemorrhagic fever Predictors: Age, hospitalization, myalgia, arthralgia (*p*-value < 0.001 for all) Limitation: Lack of further assessment by psychiatrist
Rashid et al. [15]	Cross-sectional (*n* = 480)	Higher scores of anxiety in undiagnosed cases and higher levels of depression in the recovery phase	Three groups: Diagnosed with dengue fever, in recovery, undiagnosed but fearful of contracting dengue	Beck's Depression Inventory (BDI) and Beck's Anxiety Inventory (BAI) were administered
Faiza et al. [16]	Cross-sectional (*n* = 270)	Higher association of depression, stress, and anxiety in middle-aged individuals with dengue (*p* value = 0.04, 0.05, 0.02 respectively)	Acute phase, 1 and 3 months postdengue	Predictors: Severity of fever (*p*-value = 0.05) and pain (*p*-value = 0.04) DASS-21 was administered
Lin et al. [17]	Retrospective cohort (cases = 48,884, controls = 195,536)	Increased risk of mood and anxiety disorders in all age groups	Postdengue, onset not specified	Psychotic, mood, and anxiety disorders are grouped under one category. No separate numbers Limitations: - Severity of dengue fever is not mentioned - Pathophysiology not explored - Detailed patient profile missing
Wee et al. [18]	Retrospective cohort (dengue cases = 11, 707 and COVID19 cases = 1,248,326)	Increased risk of neuropsychiatric sequelae including anxiety disorders	31–300 days postdengue	Risks of psychiatric sequelae were compared with COVID-19 population Infection was attributed to the DENV-3 serotype Limitation: Asymptomatic group was not evaluated
Shih et al. [19]	Population-based cohort (*n* = 45,334)	An increase in depression was observed in all timeframes. No elevated risk of anxiety disorder across all timeframes	- Immediate (< 3 months) - Intermediate (3–12 months) - Prolonged (> 12 months of dengue)	Sleep disorder increased in the intermediate period Outcomes identified by a minimum of one hospital admission or less than three outpatient visits Limitations: - Diagnosis of psychiatric disorders done through insurance database. No structured interviews - Not able to differentiate secondary dengue infections

**Table 4:** Mania secondary to dengue infection—key findings noted are that mania associated with dengue typically emerges during the febrile phase or within 2 weeks postinfection.

Reference	Study	Clinical features	Onset of psychiatric symptoms	Management	Remarks
Mendhekar et al. [20]	Case report	Increased activity and talk, irritability, decreased need for sleep, delusion of grandeur	Sixth day, febrile phase	Carbamazepine 600 mg/day and haloperidol 15 mg/day	Associated features: Severe headache, confused behavior and low platelet count (20000/μL), increased bleeding time

Bhatia et al. [21]	Case report	Excess talk and activity, decreased need for sleep, delusion of grandeur	Third day, febrile phase	Divalproex sodium 1500 mg/day, risperidone 6 mg/day	Associated features: Severe headache, altered consciousness, low platelet (26,000/μL), increased bleeding time

Tripathi and Mishra [22]	Case report	Increased talk, over religiosity, decreased need for sleep	Sixth day after resolution of fever	Sodium valproate 1000 mg/day, quetiapine 300 mg/day	Associated features: Low platelet (62,000/μL), increased bleeding time. Neuroimaging had no structural abnormalities

Harder et al. [23]	Case report	Increased talk, increased energy, elated mood	Not specified	Not mentioned	No prior hypomanic episodes despite antidepressant trials

Krishnan et al. [24]	Case series	Case 1: Decreased sleep, increased talk, irritability, over-religiosity, delusion of grandeur	One week after resolution	Sodium valproate 1500 mg/day and olanzapine 15 mg/day	No encephalopathy in all cases
		Case 2: Decreased sleep, increased energy and talk, increased interaction, expansive ideas	Five days after resolution	Haloperidol 10 mg/day	
		Case 3: Decreased sleep, increased talk, increased psychomotor activity, elated mood	Four days after resolution	Olanzapine 5 mg/day	

Dinakaran et al. [25]	Case report	Mania with psychotic symptoms	Within 2 weeks of dengue fever	Valproate, quetiapine, olanzapine	The role of histone deacetylase in etiopathogenesis was explored

Pariwatcharakul and Srifuengfung [26]	Case report	Lability, aggressive, decreased need for sleep, distractible, increased talk, impulsivity	Ten days after resolution	Sodium valproate 1500 mg/day, tizanidine 12 mg/day, clonidine and quetiapine 800 mg/day	Features of dengue hemorrhagic fever with shock
					The role of alpha 2 adrenergic receptor agonists in therapeutics was explored

Elavia et al. [27]	Case report	Increased activity, impulsivity, pressure of speech, decreased need for sleep, delusion of grandeur	Five days after resolution	Olanzapine 20 mg/day and lorazepam 2 mg/day	Neuroimaging had no structural abnormalities

**Table 5:** Psychosis secondary to dengue infection—key findings of psychosis are reported in both acute and recovery phases.

Reference	Study	Clinical features	Onset of psychiatric symptoms	Management	Remarks
Blum et al. [28]	Case report	Agitation, ideas of reference, visual hallucinations	Timeline could not be established	Risperidone, lorazepam, single-dose fluphenazine depot	Fever is not a prominent symptom during hospitalization. Dengue encephalitis noted
Aggarwal and Nimber [29]	Case report	Catatonia	Acute phase	Lorazepam	Medical management with antibiotics (ceftriaxone)
Kar and Prakash [30]	Case report	Suspiciousness, decreased sleep, poor self-care, delusion of persecution	Recovery phase	Olanzapine 10 mg/day, lorazepam 2 mg	
Abdullah and Bakar [31]	Case report	Delusion of persecution, auditory and visual hallucinations	Acute phase	Quetiapine titrated up to 200 mg	
Chaudhury et al. [32]	Case report	Persecutory delusions, auditory and visual hallucinations	Acute phase	Risperidone 4 mg/day	
Bhatnagar and Prasad [33]	Case report	Irritability, irrelevant talk	Recovery phase	Haloperidol 0.25 mg twice/day	Dengue shock syndrome with delirium was noted in the child. Hence, diagnosis of psychosis is uncertain due to overlapping features with delirium
Hussain et al. [34]	Case report	Agitation, altered sensorium	Third day after fever, acute phase	Haloperidol 5 mg intramuscular	Dengue encephalitis noted. Medical management with antivirals
Lin et al. [17]	Retrospective cohort study (cases = 48,884, controls = 195,536)	Increased risk of psychotic, mood, and anxiety disorders in all age groups after dengue fever	Postdengue infection, onset not specified	N/A	The severity of dengue fever is not mentioned Detailed patient profile missing Psychotic, mood, and anxiety disorders are grouped under one category

**Table 6:** Body dysmorphic disorder secondary to dengue infection.

Reference	Study	Clinical features	Onset of psychiatric symptoms	Management	Remarks
Hafi and Uvais [35]	Case report	Telogen effluvium presents as body dysmorphic disorder	Postdengue, duration not specified	Sertraline, clonazepam	

## Data Availability

All data supporting this narrative review are from previously published studies and have been cited.

## Supporting Information

Supporting Figure: Selection process of the studies for the review.
