# Supplementary material for: Psychiatric Sequelae of Dengue: A Review of the Interface
Source: J Trop Med. 2025 May 25;2025:7136558. doi: 10.1155/jotm/7136558 (PMC12127122; doi:10.1155/jotm/7136558)
Supplement: Supporting Information — Additional supporting information can be found online in the Supporting Information section. [file 7136558.f1.docx]

**Supplementary material:**

**
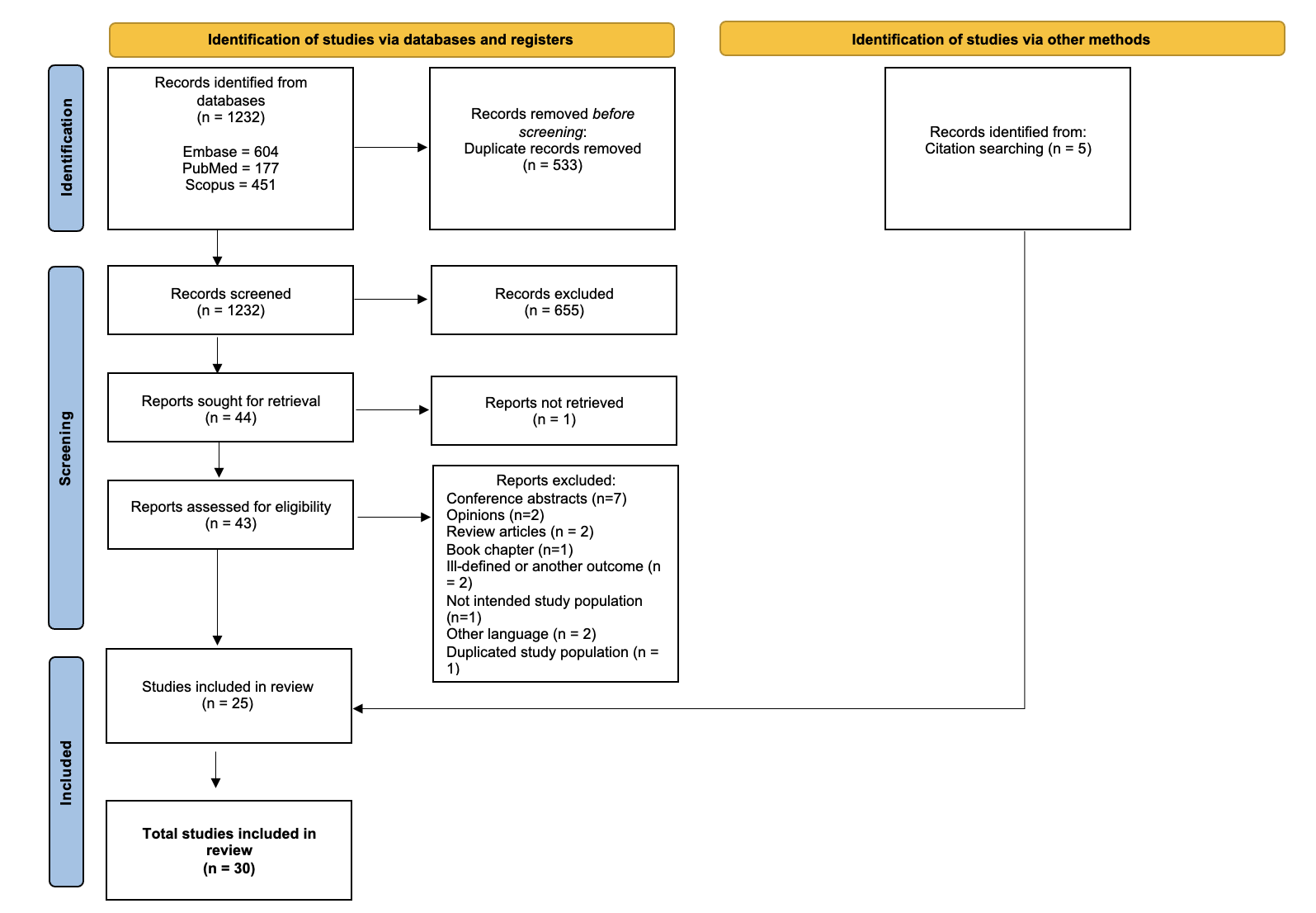
**

Supplementary Figure 1: Selection process of the studies for the review
